# First Insights Into the Biological and Physical–Chemical Diversity of Various Salt Ponds of Trapani, Sicily

**DOI:** 10.1111/1758-2229.70075

**Published:** 2025-03-21

**Authors:** Benvenuta Sonia Cusenza, Giuseppe Scelfo, Gabriella Licata, Fanny Claire Capri, Fabrizio Vicari, Rosa Alduina, Valeria Villanova

**Affiliations:** ^1^ Dipartimento STEBICEF Università degli Studi di Palermo (UNIPA) Palermo Italy; ^2^ Dipartimento di Ingegneria Università degli Studi di Palermo (UNIPA) Palermo Italy; ^3^ RESourSEAs SrL Palermo Italy

**Keywords:** halophilic microorganisms, *Halorubrum*, hypersaline, ionic composition, metabarcoding, microbial diversity

## Abstract

The salt ponds of Trapani, Sicily, represent an extreme and under‐explored ecosystem characterised by varying salinity gradients and environmental conditions. These ponds, integral to traditional salt extraction, include cold, driving, hot and crystallizer ponds, each hosting diverse microbial communities. This study aimed to explore the biological and physical–chemical diversity of 11 ponds during the salt production season in Trapani. We conducted comprehensive physical–chemical characterizations, including measurements of pH, conductivity, viscosity, density, organic carbon and ion concentration. Microbial DNA was extracted from salt pond waters and subjected to metabarcoding of 16S rRNA genes to determine the diversity of archaea and bacteria. High‐throughput sequencing revealed significant variations in microbial communities across different pond types and seasons. Cold ponds showed a higher diversity of moderately halophilic organisms, while crystallizer and feeding ponds were dominated by extreme halophiles, particularly archaeal genus *Halorubrum* and *Haloquadratum* and bacterial genus *Salinibacter*. Statistical analyses indicated that environmental parameters, especially salinity and temperature, significantly influenced microbial community composition. Our findings enhance the understanding of microbial ecology in saline environments and highlight the potential of halophilic microorganisms. This study provides a foundation for future research into the functional roles of these microorganisms and their industrial applications.

## Graphical abstract

This study unveils the diverse biological and physical–chemical characteristics of the salt ponds in Trapani, Sicily, focusing on microbial community structures and environmental parameters across varying salinities. It highlights unique microbial adaptations and the dynamic interactions shaping this Mediterranean hypersaline ecosystem.

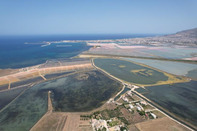

## 1 Introduction

The salt ponds of Trapani, located along the western coast of Sicily, Italy, represent a unique and dynamic ecosystem characterised by varying salinity gradients and distinct environmental conditions. These salt ponds are integral to the traditional salt extraction system, which has been practiced for centuries. The system comprises a series of interconnected ponds where seawater is gradually concentrated through solar evaporation. This method involves different types of ponds, each with specific roles and environmental conditions: cold, driving, hot and crystallizer ponds (Cipollina et al. 2012). Cold ponds are the initial stages of the salt extraction process where seawater is introduced and begins to evaporate. These ponds typically have salinity levels close to those of the original seawater. Historically, they have been used for fish farming and still today serve as the habitat for many different organisms, including diverse community of microorganisms that initiate the concentration process (De Medeiros Rocha et al. 2012). As water gradually evaporates at an average rate that depends on the salinity, it is channelled through a series of different ponds, each serving a distinct purpose. In the driving ponds, soluble heavy metals are separated and suspended organic solids settle out. As evaporation progresses, the water reaches the saturation point for calcium salts, which are released in large quantities in the hot ponds (Gómez‐Morales et al. 1996). The shallow bottoms of these ponds are close to the surface, allowing them to absorb solar radiation effectively. This absorption fosters microbial life in the carbonate‐rich sediment, creating a unique and dynamic environment (Nayak 2018). The high temperatures and increased salinity in these ponds promote the proliferation of halophilic microorganisms, which play crucial roles in nutrient cycling and biogeochemical processes (Ventosa et al. 2014). Finally, the brine reaches the crystallizer ponds, the most saline environments, where NaCl precipitation occurs. In these ponds, the concentration of total dissolved solids (TDS) encompasses a wide range of minerals, including salts of major ions (Na^+^, K^+^, Mg^2+^, Ca^2+^, Cl^−^, SO_4_^2−^) and minor inorganic ions (Sr^2+^, Li^+^, B^3+^, Br^−^, etc.) (Vicari et al. 2022).

**FIGURE 1:**
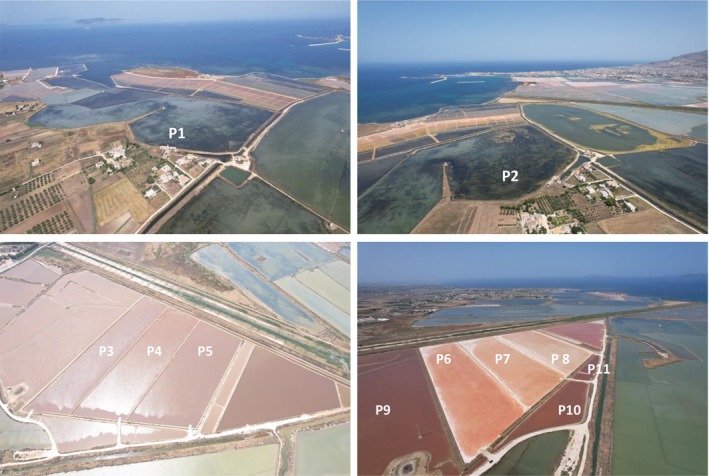
Aerial photographs of various salt ponds, including cold ponds (P1 in May and P2 in July), crystallizer ponds (P3–P5 in May and P6–P8 in July), and feeding ponds in July (P9–P11).

Here, the high concentrations of salts in these ponds allow the proliferation of halotolerant and halophilic microorganisms. Halotolerant organisms can survive across a wide range of salt concentrations, up to saturation levels, whereas halophiles require high salinity for optimal growth, thriving at concentrations of 35 g/L NaCl or higher (Oren 2008). These microorganisms, which inhabit hypersaline environments such as salt ponds, salt lakes and the Dead Sea, have evolved various chemical strategies to protect themselves from osmotic stress (Tsuzuki et al. 2011). For instance, halophiles accumulate significant amounts of carotenoids (e.g., β‐carotene, bacterioruberin, bacteriorhodopsin and salinixanthin) under high salinity and intense light conditions, which help shield their cells from UV damage and oxidative stress (Oren 2019). This accumulation of carotenoids often results in the pink‐red coloration of salt ponds during spring and summer seasons (Figure 1). Diverse taxa of halophilic microorganisms are found in these environments, spanning all three domains of life: archaea (e.g., haloarchaea), bacteria and eukaryotes (Gunde‐Cimerman et al. 2005). Among eukaryotic microorganisms, the green alga *Dunaliella salina* is particularly noteworthy. This flagellate microalga is highly studied due to its ability to accumulate substantial amounts of β‐carotene (up to 14% of its dry biomass) under extreme environmental conditions, making it one of the few microalgae utilised for industrial β‐carotene production (González et al. 2005; Lamers et al. 2008). In the domain of Archaea, haloarchaea of the class Halobacteria, are prevalent in hypersaline environments. These red‐coloured species are dominant in solar salterns where salt concentrations are at saturation levels, often coexisting with *Dunaliella salina* (Oren 2016). Among bacteria, the red bacterium *Salinibacter ruber* is the most representative bacterial member in hypersaline environments (Antón et al. 2000; Oren 2013).

Only a handful of research projects focused on the characterisation of microorganisms from Salt ponds of Trapani using both culture dependent and independent methods (Barghini et al. 2015; Huby et al. 2021; Arena et al. 2021; Villanova et al. 2021). However, an extensive study of the bacterial community that populates the different groups of Trapani's saltern ponds is missing. Previous studies employing 16S rDNA metabarcoding and metagenomics on other salt ponds, such as those in Santa Pola, Spain, and Margherita di Savoia, Italy, have demonstrated that these extreme environments host rich and diverse microbial communities (Ventosa et al. 2015; Leoni et al. 2020). These studies have identified a variety of prokaryotic populations, including archaea and bacteria However, a combination of extensive biological and physical–chemical analysis in various salt ponds is missing. The microbial diversity in these ecosystems plays a crucial role in the biogeochemical processes and contributes to the unique characteristics of the salt ponds.

In our study, we focused on the biological and physical–chemical characterisation of 11 salt ponds of Trapani during two different times within the salt production season. To comprehensively analyse the microbial communities, we employed metabarcoding of 16S rRNA genes. This approach allowed us to accurately determine the diversity and relative abundance of archaea and bacteria, across different pond types and seasonal variations. By employing a combination of microbiological and physical–chemical analyses, we aimed to elucidate the diversity and functional potential of halophilic microorganisms inhabiting these distinct pond environments. This research not only enhances our understanding of the microbial ecology of the under‐explored salt ponds of Trapani but also explores the potential of its halophilic microorganisms for various biotechnological applications. The findings from our study provide valuable insights into the complex interactions within the microbial communities and the environmental factors that influence their distribution and functionality.

## 2 Materials and Methods

### 2.1 Study Area and Sampling

The study was conducted in the salt ponds of Trapani in 2023, located along the western coast of Sicily, Italy. These ponds are part of a traditional salt extraction system comprising various types: cold ponds, driving ponds, hot ponds, and crystallizer ponds. In Sicily, the salt production season in salt ponds typically starts in late spring, around May, and extends through the summer until early autumn, usually September. The warm and dry Mediterranean climate of Sicily provides ideal conditions for salt crystallisation, with consistent sunshine and minimal rainfall during these months. In particular, sampling was performed at two different times of the salt production season (i.e., the 24th of May and the 21st of July) to capture seasonal variations in microbial communities and environmental conditions. Temperature data, estimated from the Trapani airport readings, indicated average temperatures of 20°C–23°C in May and 30°C–34°C in July (https://it.weatherspark.com/h/y/71757/2023/Condizioni‐meteorologiche‐storiche‐durante‐il‐2023‐a‐Trapani‐Italia#Figures‐Summary).

Samples were collected from 11 ponds of different types:

− Cold ponds: Represent initial stages of seawater evaporation.
− Crystallizer ponds: Represent final stages of evaporation, with the highest salinity, where salt precipitation occurs.
− Feeding ponds: Serve as intermediary basins in the evaporation process, regulating the seawater flow toward crystallizer ponds during salt extraction

The aim of the study was to compare the biological and physical–chemical compositions of three crystallizer ponds across two sampling times: May (early salt production season) and July (peak salt production season). Cold ponds were included as outgroup controls for both times, and in July as they represent the least saline environment and provide a reference point for understanding the progression of salt concentration effects. Feeding ponds were included only in July because noticeable colour changes were observed during this period (Figure 1).

Approximately 1 L of water was collected from each of the 11 ponds using sterile bottles and transported on ice to the laboratory for further analysis.

The number of ponds and samples analysed per category and time point were summarised in Table 1.

**TABLE 1:** Summary of the ponds sampled across the study, categorised by type (cold, crystallizer, feeding) and time point (May and July).

Pond type	May (*n* = ponds)	July (*n* = ponds)	Notes
Cold ponds	1	1	Used as outgroup controls
Crystallizer ponds	3	3	Main focus of statistical analyses
Feeding ponds	—	3	Added in July due to observed colour changes

### 2.2 Physical–Chemical Analysis

Environmental parameters, including pH, conductivity, density and viscosity were measured to fully characterise the salt ponds water with their physical–chemical properties.

Temperature, conductivity and pH were monitored during tests using a WTW pH/Cond 3320 Universal Multi‐parameter Portable Meter in both retentate and permeate flows. The pH probe was calibrated using a 4–7–10 standard pH buffer solutions, while the conductivity probe (WTW TetraCon 325) was calibrated using 1413 μS/cm standard solution.

Ion chromatography (IC) was used to detect the presence and concentration of major ions, that is, Na^+^, K^+^, Mg^2+^, Ca^2+^, Cl^−^, SO₄^2−^, Br^−^. For cation analysis, a Metrohm 882 compact IC plus column was used with 0.5 mM oxalic acid and 4.5 mM nitric acid solution as the mobile phase. For anion analysis, the mobile phase was constituted by a solution of 3.2 mM Na_2_CO_3_ and 1 mM NaHCO_3_ used with the Methohm 930 compact IC plus column. Samples were filtered through 0.45 μm Nylon Filter‐lab filters and diluted in ultrapure water. In addition, an inductively coupled plasma atomic emission spectrometer (ICP‐OES, Optima 2100 DV PerkinElmer) was used for the determination of boron concentration. Salt ponds waters were diluted in 2% w/w nitric acid before measurement.

Viscosity data were obtained using two Cannon‐Fenske capillary viscometer (0.4–6 Cst) provided by Xylem Analytics Germany and Ubbelohde capillary viscometer (2–10 Cst) provided by Bicasa s.r.l. while density tests were performed by weighing 50 mL of fluid in a graduated flask on a precision balance (Ohaus balance, Explorer EX 324).

TDS was measured bringing to complete evaporation 50 mL of samples in an oven (TCN 50 Plus). The adopted procedure involves heating the sample at 105°C for 8 h and further heating at 180°C for another 8 h to expel crystallisation water from the sample as well.

At the end non‐purgeable organic carbon (NPOC) was detected with Shimadzu TOC‐VCN analyser to detect the carbon contents of salt pond water. Samples were filtered through 0.45 μm Nylon Filter‐lab filters and diluted in ultrapure water.

### 2.3 Microbial Community Analysis

#### 2.3.1 DNA Extraction

About 50 mL of ponds water was filtered through 0.2 μm pore size filters (Whatman mixed cellulose ester membrane filters) using a peristaltic pump. Genome DNA was extracted from filtered ponds water using DNeasy Power Water Kit, following the manufacturer's protocols. The quality and quantity of extracted DNA were assessed using a NanoDrop 2000c spectrophotometer (Thermo Fisher Scientific, MA, USA) and agarose gel electrophoresis.

#### 2.3.2 Metabarcoding of 16S rRNA Gene

To characterise the microbial communities, we performed metabarcoding of the 16S rRNA gene for prokaryotes (bacteria and archaea) The V3–V4 region of the 16S rRNA gene was amplified using the universal primers Pro341F CCTACGGGNBGCASCAG and Pro805R GACTACNVGGGTATCTAATCC (Takahashi et al. 2016) modified to include Illumina overhang adaptors.

#### 2.3.3 PCR Amplification and Sequencing

PCR amplifications were performed in 25 μL reactions containing12.5 μL of Q5 Hot Start High fidelity 2× Master Mix, 1.25 μM of each primer and 10–80 ng of template DNA. The thermal cycling conditions were as follows: initial denaturation at 95°C for 5 min, followed by 25 cycles of 95°C for 30 s, 56°C for 30 s and 72°C for 30 s, with a final extension at 72°C for 2 min. Amplicons were purified using Kit E.Z.N.A. Cycle Pure. Equimolar amounts of purified amplicons were pooled and libraries were constructed and sequenced on an Illumina MiSeq platform in paired end 300‐bp mode read length at a commercial sequencing facility (IGA Technology Services s.r.l.).

#### 2.3.4 Bioinformatics Analysis

IGA Technology Services s.r.l analysed metabarcoding sequences based on internal pipeline. Raw sequence data was processed using QIIME pipeline (Bolyen et al. 2019). Briefly, sequences were demultiplexed, quality‐filtered and trimmed. Following the QIIME pipelines, the USEARCH algorithm (version 8.1.1756, 32‐bit) allows chimera filtering, grouping of replicate sequences; sorting sequences per decreasing abundance and Operational Taxonomic Unit (OTU) identification. Taxonomic classification was performed using the SILVA 138.1 rep set SSU 16S (https://www.arb‐silva.de/ngs/) only database for 16S rRNA gene sequences with a clustering threshold set at 97% and a minimum confidence threshold of 0.50. Rarefaction curves end‐points and normalisation of counts for diversity analysis were set to 50% of the target sequencing coverage (i.e., for 100,000 fragments a cutoff of 50,000 fragments is applied). Based on the rarefaction curve, α‐diversity metrics (Shannon and Simpson), Good's coverage index and β‐diversity (Bray–Curtis dissimilarity) metrics were calculated on a rarefied frequency‐feature table, with a minimum number of 10.000 sequences per sample, to assess the microbial diversity within and between pond types and sampling periods. Principal coordinates analysis (PCA) was performed starting from the Bray‐Curtis distance matrix, using the software package Emperor. HeatMap was implemented through the online webserver (http://heatmapper.ca/expression/) based on the 20 most abundant families (relative abundance > 1.2%), and it was generated by average link calculation using the Euclidean correlation.

METAGENassist (http://www.metagenassist.ca) was employed to distinguish microbial genus from crystallizer and Feeding ponds based on their metabolic activity. In MetagenAssist, the functional analysis framework is not strictly limited to mutually exclusive categories. A single taxon can often be associated with multiple functional categories, reflecting the diverse roles that microorganisms may play in their environment.

#### 2.3.5 Statistical Analysis

The physical–chemical analyses in the various ponds were compared by *t*‐test analysis using GraphPad 9.3.1 (Software 2365 Northside Dr. Suite 560, San Diego, CA 92108, USA). *p* values were used to quantify the variability between the various ponds. Data were considered significant for *p* values < 0.05. The biological analyses were compared by Tukey's multiple comparisons test using GraphPad 9.3.1. *p* values were used to quantify the variability between the crystallizer and feeding ponds in May and July. Data were considered significant for *p* values < 0.05.

## 3 Results

### 3.1 Physical–Chemical Characterisation of Trapani Salt Ponds

**FIGURE 2:**
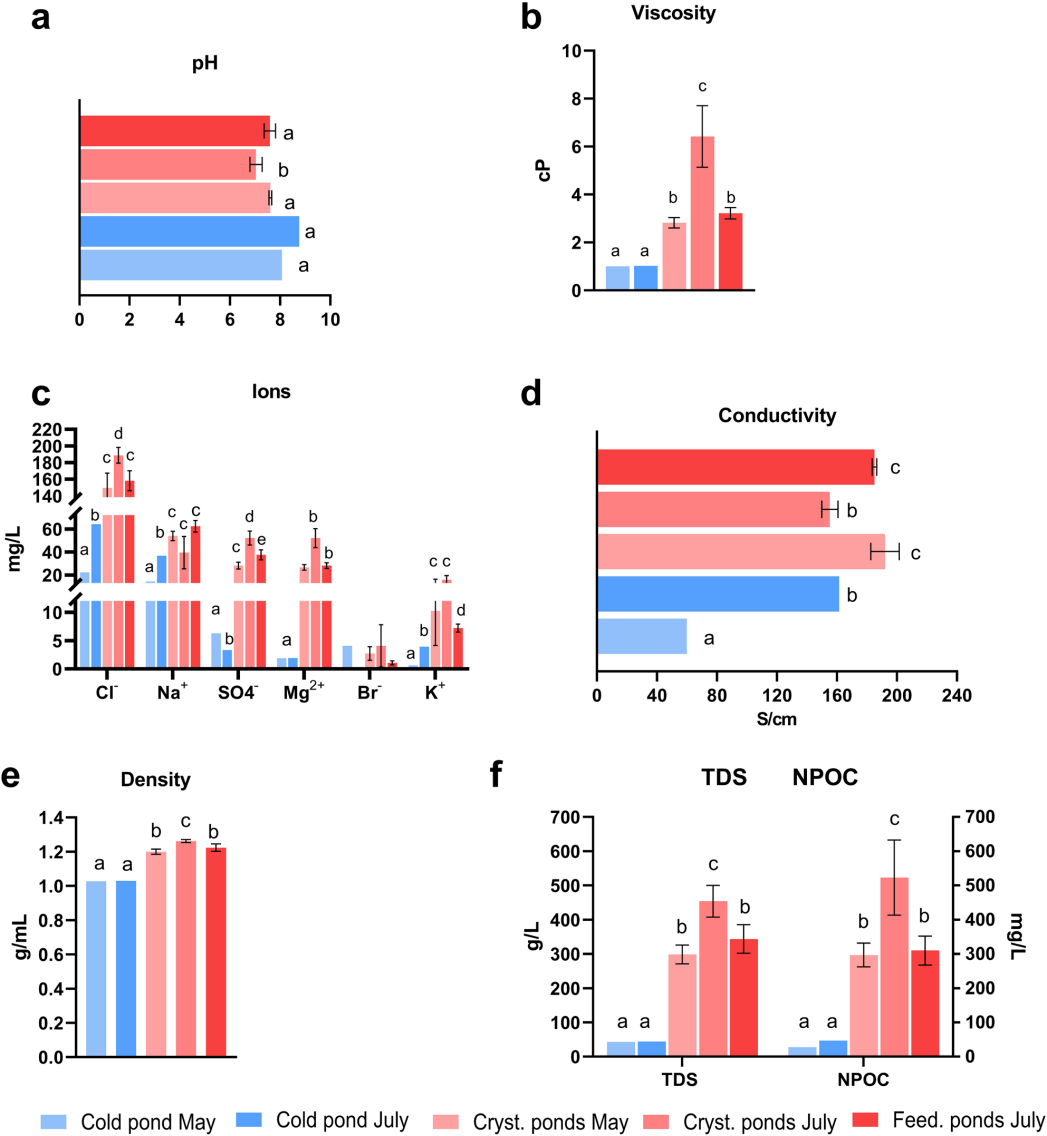
Physical–chemical characterisation of different salt ponds (cold, crystallizer and feeding ponds) at different seasons (May, July). (a) pH value, (b) viscosity, (c) ions, (d) conductivity, (e) density and (f) total dissolved solids (TDS) and non‐purgeable organic carbon (NPOC) analysis. Graph shows data as mean ± standard deviation (*n* = 3) from ponds of the same type, except for cold ponds, where single samples were collected at each time point and used as outgroup controls. Different letters (a, b, c, d) denote significant differences among ponds (*t*‐test, *p* < 0.05).

Physical–chemical characterisation of water samples collected from cold, feeding, and crystallizer ponds of Trapani during May and July were shown in Figure 2.

The pH trend is less predictable than the other parameters here shown, having similar values in the different ponds, ranging between 7 to 8.5. However, a slight but significant decrease in pH was observed in the crystallizer ponds in May compared to the other samples (Figure 2a). In contrast, the variation in viscosity was much more pronounced than the other parameters recorded across the different ponds, ranging from approximately 1 to 8 cP. Notably, the crystallizer ponds in July exhibited significantly higher viscosity compared to the other ponds. Conversely, the viscosity in the cold ponds remained around 1 cP (Figure 2b).

The IC analysis identified the major ionic constituents (Cl^−^, Na^+^, SO₄^2−^, Mg^2+^, Br^−^, K^+^ and Ca^2+^) in the different types of ponds. Cold ponds showed generally lower ion concentrations compared to the other ponds, with a slight increase from May to July in Cl^−^ and Na^+^ concentrations, likely due to evaporation caused by seasonal temperature increases. Crystallizer ponds in July exhibited the highest ion concentrations, with notable levels of Cl^−^, SO₄^2−^, Mg^2+^ and K^+^. However, the Na^+^ level in these ponds was lower than in other high‐salinity ponds. Feeding ponds in July showed similar ion concentrations to crystallizer ponds in May (Figure 2c). Additionally, B^3+^ and Ca^2+^ were only detected in trace amounts, as reported in Data S1.

The electrical conductivity of water in different ponds was significantly lower in cold pond in May compared to the other ponds. The highest value of conductivity was showed in both crystallizer ponds in May and in feeding ponds in July (Figure 2d).

The density of water was affected by seasons and type of ponds. In particular, it was higher in both crystallizer and feeding ponds compared to cold ponds due to the higher concentration of salts in these ponds (Figure 2e). Finally, it was determined the concentrations of TDS and NPOC in various ponds. Both values were significantly higher in both crystallizer and feeding ponds compared with cold ponds. The highest value of TDS and NPOC were reported in crystallizer pond of July (Figure 2f). These analyses provided insights into the physical–chemical conditions that could influence microbial diversity.

### 3.2 Bacterial Community Analysis of Trapani Salt Ponds at Phylum Level

**FIGURE 3:**
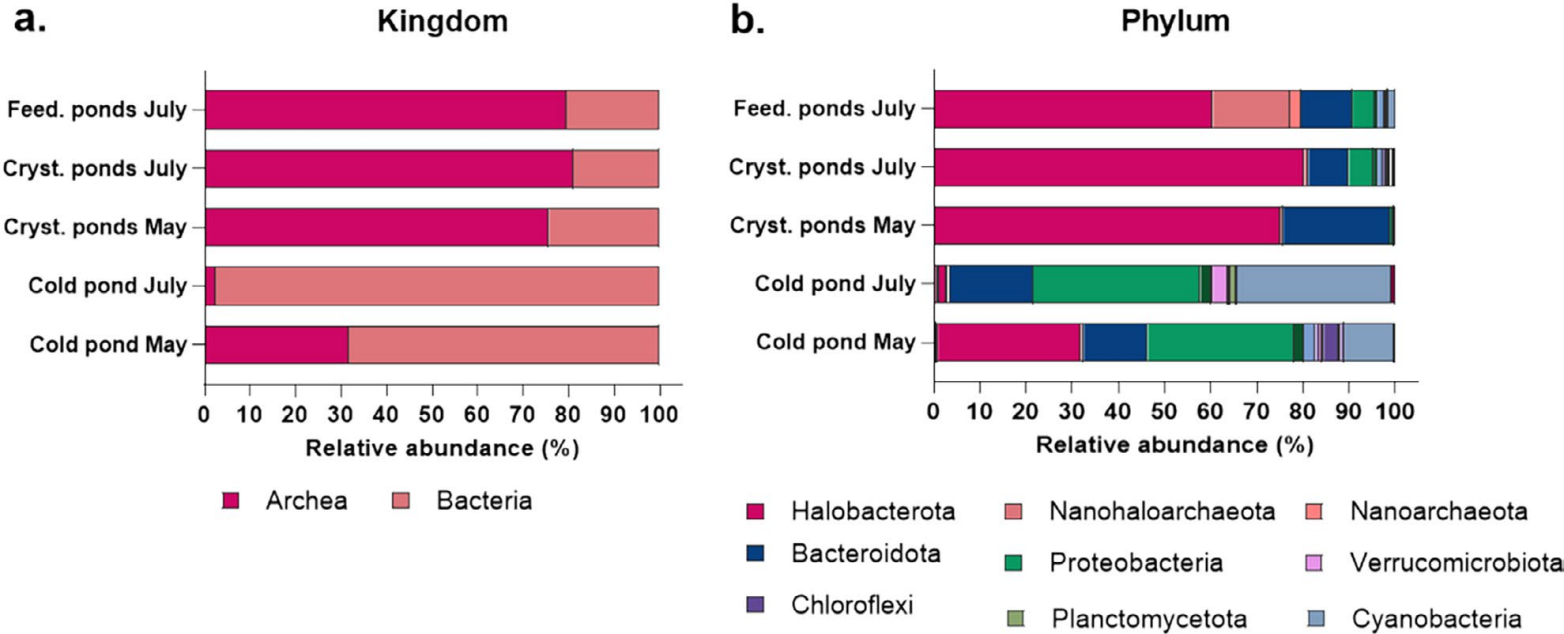
Bacterial community analysis at (a) kingdom and (b) phylum level in different salt ponds (cold, crystallizer and feeding ponds) in different seasons (May, July). Graph shows data as mean (*n* = 3) from ponds of the same type, except for cold ponds, where single samples were collected at each time point and used as outgroup controls.

The bacterial community analysis allowed to determine the pattern of archaea and bacteria in the different ponds and seasons. Both crystallizer and feeding ponds showed a comparable high concentration of archaea composition (ranging from 75.7%–81%) with no significant statistical difference observed between these groups (Data S2). Cold ponds showed a lower relative abundance of archaea, with 31.6% in May and 2.4% in July compared to a higher concentration of the bacterial community ranging from 68.4% to 97.6% across the same time points (Figure 3a). Further comparison of archaeal composition at the phylum level revealed the presence of three distinct phyla (61.3% ± 4.49% of Halobacteriota, 15.49% ± 7.27% of Nanohaloarchaeota and 2.67% ± 1.01% of Nanoarchaeota) in the feeding ponds, whereas only one phylum (Halobacteriota) was identified in the crystallizer ponds during both May (74.1% ± 6.14%) and July (79.27% ± 7.49%). Statistical analysis revealed no significant differences in the relative abundance of Nanohaloarchaeota between the two crystallizer ponds (May vs. July, *p* = 0.4937), but their abundance was significantly higher in feeding ponds compared to both crystallizer ponds (*p* = 0.0129 for May; *p* = 0.0108 for July, Data S2).

Moreover, Bacteroidota was the most abundant bacterial phylum in all high‐salinity ponds. Specifically, the highest percentage was detected in crystallizer ponds May (24.02% ± 5.41%), followed by feeding ponds (11.58% ± 3.28%) and crystallizer ponds July (9.48% ± 3.05%). Statistical comparisons highlighted significant differences in Bacteroidota abundance between crystallizer ponds in May and July (*p* = 0.0159) and between crystallizer ponds in May and feeding ponds (*p* = 0.022). However, no significant differences were observed between crystallizer ponds in July and feeding ponds (*p* = 0.0668, Data S2).

A greater diversity of bacterial phyla was instead observed in the cold ponds, with Bacteroidota (13.87% in May and 9.48% in July), Proteobacteria (31.67% in May and 36.23% in July), and Cyanobacteria (10.82% in May and 33.28% in July) being predominant. (Figure 3b, Data S3).

### 3.3 Bacterial Community Analysis of Trapani Salt Ponds at Family Level

**FIGURE 4:**
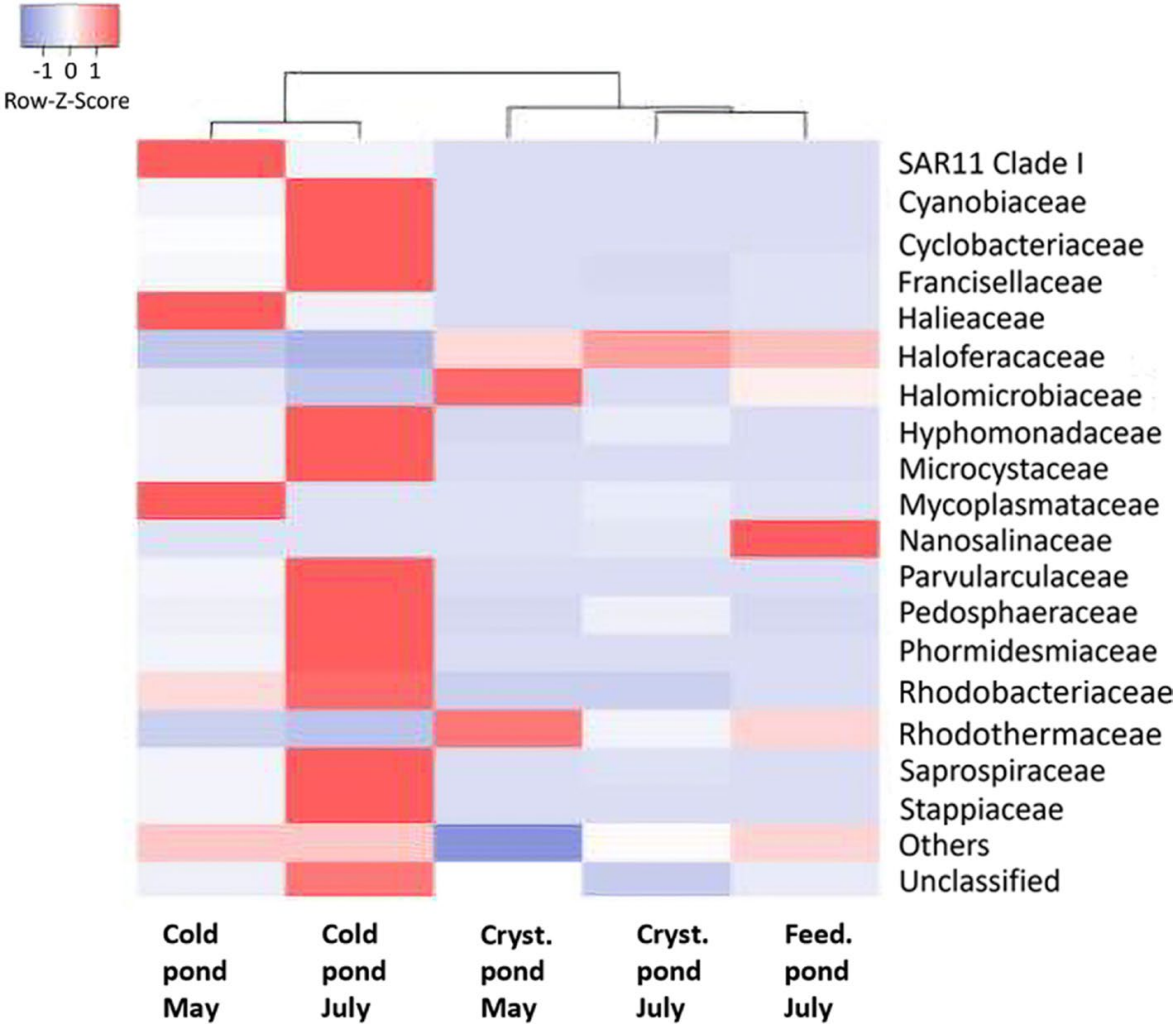
The heatmap represents the relative abundance of 20 most abundant microbial families across different pond types (cold, crystallizer and feeding) and time points (May and July). Graph shows data as mean (*n* = 3) from ponds of the same type except for cold ponds, where single samples were collected at each time point and used as outgroup controls. The colour scale indicates relative abundance, with red representing higher abundance and blue representing lower abundance. The dendrogram displayed above the heatmap illustrates the hierarchical clustering of microbial communities across different pond types and sampling times.

The dendrogram analysis reveals a clear separation between cold ponds and the other groups of ponds. Specifically, the cold ponds, sampled in both May and July, form a distinct cluster, indicating a unique microbial community composition compared to the other ponds. Another cluster is characterised by the crystallizer and feeding ponds sampled in July, suggesting that the microbial communities in these ponds are more similar to each other. This clustering likely reflects the influence of higher temperatures and increased salinity on microbial community structure. Crystallizer and feeding ponds show the presence of specialised microbial community, with certain halophilic families being highly predominant. In particular, both time points of crystallizer and feeding ponds show a predominance of specific haloarchaeal families such as Haloferacaceae and Halomicrobiaceae (Figure 4). Notably, Haloferacaceae is highly abundant, constituting 74.93% ± 9.71% in the crystallizer ponds in July, 53.08% ± 17.74% in the crystallizer ponds in May and 52.65% ± 4.76% in the feeding ponds. Additionally, the Halomicrobiaceae family is present in significant amounts, accounting for 20.50% ± 11.51% in the crystallizer ponds in May, 6.76% ± 0.29% in the feeding ponds, and 3.01% ± 1.33% in the crystallizer ponds in July. Moreover, there was a higher relative abundance of the haloarchaeal family Nanosalinaraceae (15.49% ± 7.27%) in feeding ponds compared to other pond types. Statistical analysis confirmed significant differences in the relative abundance of Nanosalinaraceae between the feeding ponds and both crystallizer ponds (*p* = 0.0177 for May and *p* = 0.0221 for July), while no significant difference was observed between the two crystallizer time points (*p* = 0.4937, Data S2). The extreme halophile bacterial family Rhodothermaceae is also notably present, constituting 21.65% ± 4.58% in the crystallizer ponds in May, 10.56% ± 2.96% in the feeding ponds, and 8.16% ± 2.72% in the crystallizer ponds in July. Statistical analysis indicated significant differences between the crystallizer ponds in May and July (*p* = 0.0155) and between the crystallizer ponds in May and the feeding ponds (*p* = 0.0156). However, no significant difference was observed between the crystallizer ponds in July and the feeding ponds (*p* = 0.188, Data S2). Overall, the microbial composition in crystallizer ponds shows relative stability from May to July, suggesting that high salinity has a more significant influence on microbial communities than temperature. Cold ponds exhibit the highest family‐level diversity, indicating a less selective environment and a more varied microbial ecosystem. In particular, cold ponds in May were dominated by a variety of families of marine bacteria including SAR11 clade 1 (8.36%), Halieaceae (2.27%) and Mycoplasmataceae (1.96%; Giovannoni 2017; Palladino et al. 2021; Suzuki et al. 2019). Moreover, in cold ponds from July, there was a notable increase in the following bacterial families: Cyanobiaceae (5.23%), Cyclobacteriaceae (1.39%), Francisellaceae (1.98%), Hyphomonadaceae (2.64%), Microcystaceae (2.72%), Parvularculaceae (2.22%), Pedosphaeraceae (1.98%), Phormidesmiaceae (1.94%), Saprospiraceae (10.55%) and Stappiaceae (3.57%, Data S3). This increase could be attributed to the specific environmental conditions prevalent in these ponds particularly the interplay of temperature and salinity. This heatmap provides valuable insights into the microbial diversity and dynamics across different environmental conditions, highlighting the adaptability and specialisation of microbial communities in extreme habitats.

### 3.4 Genus‐Level Relative Abundance and Metabolism Analysis in High‐Salinity Ponds

**FIGURE 5:**
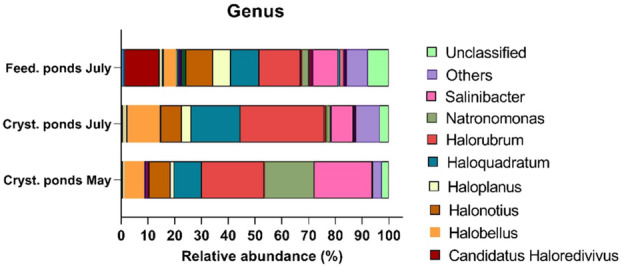
Bacterial community analysis at genus level in crystallizer, and feeding ponds in different seasons (May, July). Graph shows data as mean (*n* = 3) from ponds of the same type.

Relative abundance of various microbial genera in crystallizer and feeding ponds sampled in May and July were shown in Figure 5. The analysis reveals distinct patterns in the microbial community composition across different pond types and sampling periods. Data from cold ponds were not shown because exhibited a high percentage of unknown genera (Data S3).

In both crystallizer and feeding ponds, the microbial community is predominantly composed of the archaeal genus *Halorubrum*, which constitutes the majority of the population. Specifically, *Halorubrum* accounts for the highest proportion in crystallizer ponds in May (23.23% ± 6.33%), followed by crystallizer ponds (31.32% ± 8.63%) and feeding ponds in July (15.45% ± 2.34%). Additional genera identified include the bacteria *Salinibacter*, with the highest concentration in crystallizer ponds in May (21.53% ± 4.5%), followed by feeding ponds (9.3% ± 3.58) and crystallizer ponds in July (8.13% ± 2.7). Statistical analysis revealed significant differences in *Salinibacter* abundance between crystallizer ponds in May and July (*p* = 0.0148) and between crystallizer ponds in May and feeding ponds (*p* = 0.01). However, there was no significant difference between crystallizer ponds in July and feeding ponds (*p* = 0.6685; Data S2).

*Natronomonas* showed the highest concentration in crystallizer ponds in May (18.44% ± 12.27%), followed by feeding ponds in July (2.4% ± 1.2%) and the lowest in crystallizer ponds in July (1.23% ± 0.97%). *Haloquadratum* had the highest concentration in crystallizer ponds in July (18.32% ± 5.94%), followed by feeding ponds in July (10.61% ± 5.51%) and the lowest in crystallizer ponds in May (10.26% ± 4.83%). Statistical analysis confirmed significant differences in *Haloquadratum* abundance between crystallizer ponds in May and July (*p* = 0.0114), while no significant differences were observed between other groups (Data S2). *Halobellus* was most abundant in crystallizer ponds in July (11.96% ± 0.57%), followed by crystallizer ponds in May (7.4% ± 0.18%), and the lowest concentration in feeding ponds in July (4.45% ± 3.03%). Statistical analysis revealed a significant difference in *Halobellus* abundance between crystallizer ponds in May and July (*p* = 0.0039), while differences between other groups were not statistically significant (Data S2). Finally, in the feeding ponds other genera such as the archea *Haloplanus* (6.57% ± 1.28%) and *Candidatus Haloredivivus* (12.74% ± 5.34%) were more prominent compared to other samples (Figure 5, Data S3). These variations highlight the diverse microbial communities present in different pond types and seasons.

**FIGURE 6:**
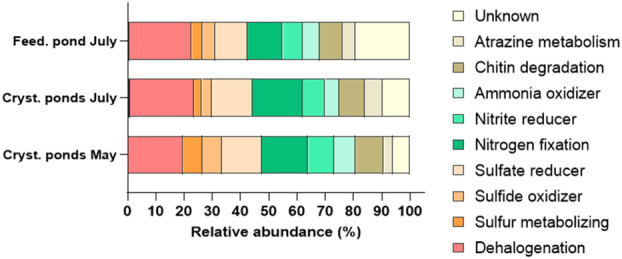
Hypothetical metabolism of Bacterial community at genus level in crystallizer, and feeding ponds in different seasons (May, July). Graph shows data as mean (*n* = 3) from ponds of the same type.

The relative abundance of various microbial functional groups in high‐salinity ponds at different times is showed in Figure 6. Both crystallizer and feeding ponds were dominated by dehalogenation (19.62% ± 1.96% in crystallizer ponds in May, 22.68% ± 1.84% in crystallizer ponds in July, and 22.13% ± 0.32% in feeding ponds), nitrogen fixation (16.4% ± 0.86% in crystallizer ponds in May, 17.8% ± 2.5% in crystallizer ponds in July and 12.49% ± 1.57% in feeding ponds), and sulfate reduction (14.23% ± 0.73% in crystallizer ponds in May, 14.46% ± 1.96% in crystallizer ponds in July and 11.42% ± 0.22% in feeding ponds). Notable concentrations of sulfate reducers and chitin degradation activities were detected across all analysed ponds. In particular, higher concentrations of sulfate reducers (14.23% ± 0.73%) and sulfide oxidizers (6.84% ± 1.36%) were detected in crystallizer ponds in May. Additionally, a large number of unknown metabolic functions were detected in feeding ponds in July, which could indicate the presence of potentially novel or less‐characterised microbial activities in these ponds (Data S4).

### 3.5 α and β‐Diversity Analysis

**FIGURE 7:**
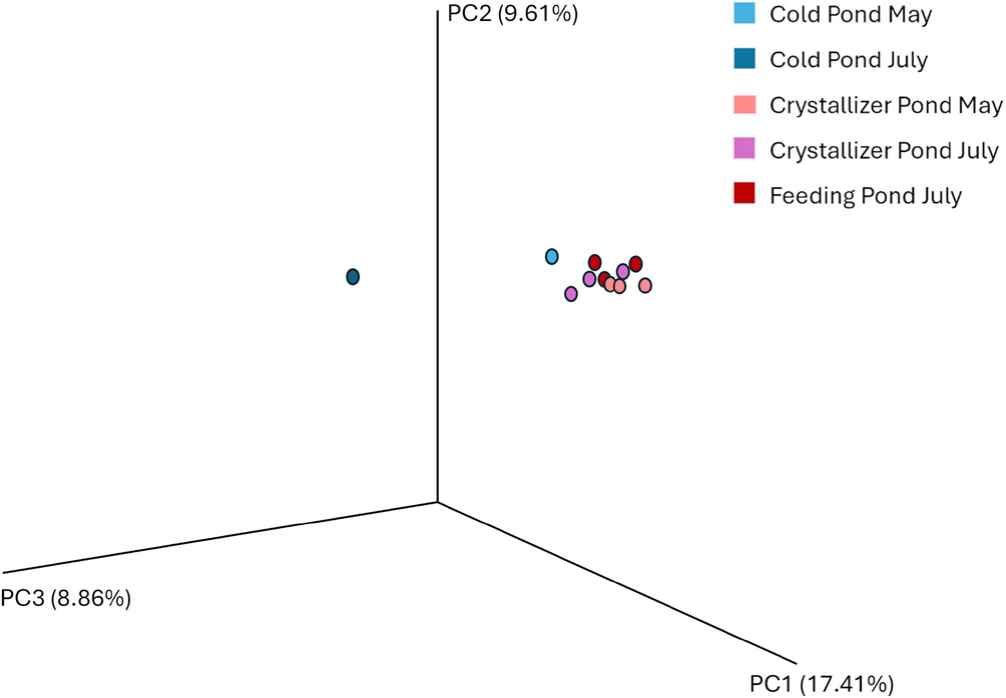
PCA analysis in cold, crystallizer, and feeding ponds in different seasons (May, July).

The PCA plot highlights clear distinctions in microbial community composition between cold ponds and high‐salinity ponds. Cold pond from May and July forms separate clusters indicating a seasonal shift in the microbial community as was for family composition (Figure 4). Crystallizer and feeding ponds cluster closely together exhibit more stability and similarity in their microbial communities over the sampling period (Figure 7).

Α‐diversity metrics for various salt ponds sampled in May and July are shown in Table 2, highlighting the number of OTU and two indices: Simpson's Index and Shannon's Index. These indices provide insights into the diversity and of the microbial communities in each pond. Simpson's Index values close to 1 indicate a high diversity across all ponds. Cold pond of May (0.99) showed the highest Simpsons Index followed by cold pond (0.98) and feeding pond of July (0.96–0.98) indicating higher biodiversity in these ponds compared to crystallizer ponds (0.91–0.97). Shannon Index was characterised by higher variations within the different ponds (i.e., 5.3–8.07). In particular, Both Shannon's and Simpson's Index values suggest that cold ponds (especially in May) have the highest diversity, whereas crystallizer ponds show lower diversity (Table 2).

**TABLE 2:** A‐diversity in cold, crystallizer and feeding ponds in different seasons (May, July).

Sample	Pond's number	Number of OTU	Simpson's index	Shannon's index
Cold pond May	P1	1483	0.99	8.07
Cold pond July	P2	518	0.98	7.48
Cryst. ponds May	P3	554	0.95	5.81
	P4	561	0.94	5.54
	P5	1031	0.95	5.69
Cryst. pond July	P6	571	0.91	5.48
	P7	727	0.92	5.33
	P8	712	0.97	6.81
Feed. pond July	P9	897	0.98	6.68
	P10	550	0.98	6.91
	P11	970	0.96	6.32

## 4 Discussion

The present study aimed to investigate the seasonal variations in the biological and physical–chemical composition of crystallizer ponds in the salt ponds of Trapani, comparing them with cold ponds and feeding ponds. This research complements earlier works by demonstrating how seasonal changes influence microbial community composition and activity in unique habitats like salt ponds (Benlloch et al. 2002; Ventosa et al. 2014; Leoni et al. 2020).

The analysis of physical–chemical parameters revealed significant seasonal variations across different pond types. In particular, it can be seen that by moving to the most concentrated feeding and crystallizer ponds, the pH decreases slightly due to the precipitation of calcium carbonate (Gómez‐Morales et al. 1996; Nayak 2018). Due to its low solubility in water, this salt is the first to precipitate within the brine, causing a slight acidification of the solution. In fact, high‐salinity ponds were characterised by a conspicuous decrease in the concentration of Ca^2+^, which was present only in trace amounts or not at all (Data S1). For this reason, respecting the same trend, a slightly lower pH value (7–7.6) was found in the crystallizer and feeding basins than in the other basins (Figure 2a) confirming previous findings (Apriani et al. 2018). These changes were accompanied by shifts in TDS (Figure 2f). Du et al. (2019) demonstrated a correlation between pH and TDS, showing how the evaporation rate, which affects the TDS content, also influences pH. When halite begins to precipitate, the proportion of strong acid‐weak base or strong base‐weak acid salts increases, leading to hydrolysis that regularly changes the pH of brine systems (Wu et al. 2012). Consequently, here it was confirmed that higher TDS content corresponded in a lower pH value (Figure 2a,f).

The influence of TDS concentration on viscosity also needs to be taken in account. This relationship is likely linked to changes in ionic composition, particularly the increased Mg^2+^/Na^+^ and SO₄^2−^/Cl^−^ ratios, as suggested by Karcz and Zak (1987) (see Figure 2d). The changes in these ionic ratios result from the saturation of sodium chloride, which stabilises the concentrations of Na^+^ and Cl^−^. Meanwhile, during evaporation in the crystallizer ponds, the concentrations of Mg^2+^ and SO₄^2−^ continue to rise below their saturation points to maintain the quality of the table salt produced. Consequently, as shown by Zohdy et al. (2013), higher Mg^2+^ concentrations correlate with lower Na^+^ concentrations in NaCl‐saturated ponds, as observed in both the crystallizer and feeding ponds (Figure 2d). Notably, both crystallizer and feeding ponds exhibited marked increases in viscosity (Figure 2b) and conductivity (Figure 2e) compared with cold ponds, consistent with the expected evaporation processes in these ponds during the warmer months (Murray 2004). Electrical conductivity is also strongly influenced by (i) TDS in solution and (ii) the ionic mobility of specific ions in solution (Nayak 2018). For this reason, crystallizer ponds in May and feeding ponds in July showed similar TDS and conductivity value (Figure 2d,f). Finally, significantly shifts in NPOC levels were detected across the various ponds, reflecting the complex interplay between microbial activity and environmental conditions. Previous studies have also reported similar trends, where salinity and temperature increase lead to enhanced microbial metabolism and organic matter production in hypersaline environments (Oren 2002; Ventosa et al. 2015). The crystallizer ponds in July, also displayed the highest viscosity and NPOC, suggesting an active microbial community influenced by the combination of higher temperatures and salinity. This finding can be attributed to the export of extracellular polymeric substances (EPS), as already shown in the salt ponds of Trapani (de Philippis et al. 1998). Additionally, the increase in viscosity can be explained by the production of glycerol by *Dunaliella salina*. *Dunaliella salina* produces glycerol as an osmoprotectant in response to high salinity, helping to balance the osmotic pressure within the cells. This glycerol and its derivatives can be released into the environment and subsequently utilised by other heterotrophic microorganisms as a carbon and energy source, further influencing the microbial community dynamics and contributing to the overall organic carbon pool (Oren, 2016).

Microbial community analysis demonstrated distinct patterns in community composition between the different pond types and seasons. 16S rDNA metabarcoding analysis showed a general trend of the relative increase in archaeal composition and decrease in bacterial diversity with the increase in salinity (Figure 3a) confirming previous results (Oren 2002). In particular, crystallizer ponds, both in May and July, were dominated by halophilic taxa such as the archaeal phylum Halobacteriota and the bacterial phylum Bacteroidota, indicating their adaptation to high‐salinity environments (Figure 3b). This dominance of halophiles is consistent with previous findings in similar hypersaline environments (Podell et al. 2014; Rosselló‐Móra et al. 2003). Feeding ponds showed similar phyla of crystallizer with the exception of the increase in the archaea Nanoarchaeota. Nanoarchaeota, a recently described phylum within the Archaea domain, is characterised by its members being small, obligate symbionts or parasites. These organisms have been predominantly found in hot springs and hydrothermal vents, but their presence in hypersaline environment has also been reported (Narasingarao et al. 2012). The cold ponds, serving as controls, showed a higher relative abundance of unclassified genera, particularly in July, highlighting the presence of less‐characterised microbial taxa in these environments. This finding underscores the need for further exploration and characterisation of microbial diversity in these systems. In high‐salinity ponds, instead, it was possible to identify a large number of taxa at genus level (Figure 5). These ponds are particularly rich in members of the haloarchaeal *Halorubrum* confirming previous results in crystallizer croatian ponds (Pašić et al. 2007). The second most representative in this study was the haloarchaeal genus *Haloquadratum*, which has been one of the main inhabitants in various salt ponds (Oren 2002; Burns et al. 2007). Moreover, this genus is particularly indicative of Mediterranean hypersaline systems (Clark et al. 2017). Finally, *Natronomonas* was highly present as a haloarchaeal genus in crystallizer ponds, but only in low abundance in feeding ponds. Different relative abundances of *Natronomonas* have also been observed in various ponds depending on the salinity level of other salt ponds in the Mediterranean Sea (Leoni et al. 2020).

Moreover, in high salinity ponds it was found *Salinibacter* as the main component of bacterial community (9%–20%); similar relative abundance of this bacteria was found in the salt pond of Santa Pola (Antón et al. 2000). Moreover, the feeding ponds, although have high similarity in physical chemical parameters (Figure 2a–f), showed some peculiarity in the bacterial composition at the genus level (Figure 5). In these ponds were found about 13% of *Candidatus Haloredivivus*, the same taxon was found in intermediate salinity salt ponds in Spain (Ghai et al. 2011). These findings can partially explain the differences in colour between feeding and crystallizer ponds (Figure 1). Additionally, also the presence of high concentration of *Dunaliella salina* , as observed under the optical microscope (data not shown), may contribute to the colour variations in these ponds. Further metabarcoding analysis of 18S rDNA in the various ponds would be needed to confirm this hypothesis.

The PCA (Figure 7) and hierarchical clustering (Figure 4) further elucidated the separation of microbial communities based on pond type and season. PCA shows a clear separation of cold ponds from the other groups emphasising the unique ecological niche occupied by these ponds, characterised by lower salinity and more diverse microbial taxa (Table 2). In contrast, the clustering of crystallizer and feeding ponds from July reflects the convergence of microbial communities under extreme environmental conditions, supporting the hypothesis that these ponds share similar selective pressures during the peak evaporation season (Benlloch et al. 2002; Ghai et al. 2011). In accordance with this result, the dendrogram revealed distinct clustering of cold ponds away from the crystallizer and feeding ponds, with the latter two groups forming a cohesive cluster in July. This pattern suggests that the increased temperature and salinity during the summer months exert a homogenising effect on the microbial communities in crystallizer and feeding ponds, likely driven by selective pressures favouring halophilic and thermophilic microorganisms (Rodríguez‐Valera et al. 1985).

Overall, most of the hypothesized microbial functions were related to coping with increased salinity and temperature (Figure 6). For example, dehalogenation, nitrite reduction, and nitrogen fixation are particularly important under these conditions for managing oxidative stress and maintaining cellular integrity (Empadinhas and da Costa 2008; Oren 2002). Furthermore, the revealed microbial metabolic functions are involved in nutrient recycling in this extreme environment. The consistent presence of chitin degradation across the samples indicates the putative role of halophile microorganisms in recycling organic matter. This function is crucial for breaking down chitin from dead organisms, thereby contributing to nutrient cycling in the ecosystem (Gooday 1990). The presence of specific microbial functions such as sulfate reduction aligns with findings from other hypersaline environments, where such processes are essential for maintaining sulfur cycles under extreme conditions (Canfield et al. 2005).

## 5 Conclusions

In conclusion, seasonal shifts in temperature and salinity play a crucial role in shaping the microbial communities and physical–chemical properties of salt ponds. This study, the first of its kind in the salt ponds of Trapani, contributes significantly to our understanding of the ecological dynamics in this hypersaline environment. Our findings provide a foundation for further investigations into the functional implications of microbial community changes and highlight the importance of seasonal variations in these ecosystems. Additionally, the discoveries from this work can be used to improve the isolation of industrially relevant microorganisms (León et al. 2014). Further studies on the eukaryotic microorganism community and organic carbon dynamics can enhance our understanding of the interactions among these microorganisms in this extreme environment. Overall, our study underscores the dynamic nature of these ecosystems and their responsiveness to environmental fluctuations, paving the way for future research on the functional roles of the identified microbial taxa and their contributions to biogeochemical cycles in salt pond ecosystems.

## Author Contributions

**Benvenuta Sonia Cusenza:** methodology, writing – review and editing, investigation, data curation, software. **Giuseppe Scelfo:** methodology, data curation, writing – review and editing, investigation. **Gabriella Licata:** methodology, writing – review and editing, investigation. **Fanny Claire Capri:** methodology, writing – review and editing. **Fabrizio Vicari:** writing – review and editing, supervision. **Rosa Alduina:** writing – review and editing, supervision. **Valeria Villanova:** conceptualization, funding acquisition, writing – original draft, writing – review and editing, formal analysis, validation, supervision, project administration, data curation, methodology.

## Conflicts of Interest

The authors declare no conflicts of interest.

## Supporting information


Data S1.

Data S2.

Data S3.

Data S4.

## Data Availability

The data that supports the findings of this study are available in the Supporting Information of this article.
